# A New Theoretical Method for Studying Effects of Microstructure on Effective Thermal Conductivity of Vermicular Graphite Cast Iron

**DOI:** 10.3390/ma16062158

**Published:** 2023-03-07

**Authors:** Ailong Jiang, Anchen Shao, Lin Song, Minghao Hua, Hongliang Zheng, Xiaofu Zhang, Xuelei Tian, Xiaohang Lin

**Affiliations:** 1 Key Laboratory for Liquid-Solid Structural Evolution and Processing of Materials, Ministry of Education, Shandong University, Jinan 250061, China; 2 State Key Laboratory of Engine Reliability, Weichai Power Co., Ltd., Weifang 261001, China; 3 Shandong Laboratory of Yantai Advanced Materials and Green Manufacture, Yantai 264000, China; 4 School of Materials Science and Engineering, Harbin Institute of Technology, Harbin 150001, China

**Keywords:** vermicular graphite cast iron, microstructure, effective thermal conductivity, unit cell model, numerical calculation

## Abstract

To provide the basis for thermal conductivity regulation of vermicular graphite cast iron (VGI), a new theoretical method consisting of shape interpolation, unit cell model and numerical calculation was proposed. Considering the influence of the graphite anisotropy and interfacial contact thermal conductivity (ICTC), the effective thermal conductivity of a series of unit cell models was calculated by numerical calculation based on finite difference. The effects of microstructure on effective thermal conductivity of VGI were studied by shape interpolation. The experimental results were in good agreement with the calculated ones. The effective thermal conductivity of VGI increases in power function with the decrease in graphite shape parameter, and increases linearly with the increase in graphite volume fraction and thermal conductivity of matrix. When the graphite volume fraction increases by 1%, the thermal conductivity of nodular cast iron increases by about 0.18 W/(m·K), while that of gray cast iron increases by about 3 W/(m·K). The thermal conductivity of cast iron has the same sensitivity to the thermal conductivity of matrix regardless of the graphite shape parameter. The thermal conductivity of matrix increased by 15 W/(m·K) and the thermal conductivity of cast iron increased by about 12 W/(m·K). Moreover, the more the graphite shape deviates from the sphere, the greater the enhancement effect of graphite anisotropy on thermal conductivity than the hindrance effect of interface between graphite and matrix. This work can provide guidance for the development of high thermal conductivity VGI and the study of thermal conductivity of composites containing anisotropic dispersed phase particles with complex shapes.

## 1. Introduction

Vermicular graphite cast iron (VGI) has become a promising material for new generation high power diesel engine castings, such as cylinder blocks and heads, due to its high specific strength and excellent comprehensive performance of mechanical and thermal conductivity compared with gray cast iron [1,2,3,4,5,6,7,8]. Recently, the properties of VGI, especially its thermal conductivity, have attracted much attention. High thermal conductivity contributes to rapid heat transfer and prevents thermal mechanical fatigue, deformation and thermal cracking, so as to ensure the application of VGI cylinder block and cylinder head under complex or harsh conditions [9,10,11,12]. It is helpful to realize the effective regulation of thermal conductivity that understand the effects of different microstructure characteristics on the thermal conductivity of VGI. However, it still remains unclear because of the difficulty of stable preparation of VGI and the complexity of the shape of vermicular graphite.

At present, the methods including statistical analysis and numerical simulation are usually used to investigate the influence of microstructure on VGI thermal conductivity. Furthermore, VGI can be regarded as an in situ composite made up of matrix and vermicular graphite [13,14,15]. Therefore, the theoretical approaches for the thermal conductivity of composites can be used to study the thermal conductivity of VGI. Statistical analysis (usually regression analysis) of a large number of experimental data can accurately obtain the positive/negative influence and weight of each variable (such as composition, microstructure, etc.) on thermal conductivity of VGI [16,17]. However, it is usually impossible to control complete independence between multiple variables in experiments. Consequently, it is difficult to intuitively study the effect of a single independent variable. The numerical simulation method can realize the control of a single microstructure variable. Previous studies, combining with experiments and 2-D or 3-D finite element analysis, reported the effects of vermicularity [18], pearlite content [19], interfacial contact thermal conductivity (ICTC) [20] and spatial connectivity [21,22] on the thermal conductivity of VGI, respectively. Nevertheless, finite element modeling of vermicular graphite is difficult considering graphite anisotropy. The theoretical approaches for the thermal conductivity of composites, including the effective medium approximation (EMA) and unit cell model [23], are suitable methods to study the thermal conductivity of cast iron [12]. Hitherto, some researchers have modified the relevant theories and models of thermal conductivity of composites and applied them to the study of thermal conductivity of cast iron. The Maxwell model is the most popular of EMA [24], which could be applied to study the thermal conductivity of nodular cast iron [25]. On the basis of the Maxwell model, EMA has undergone the expansion and correction of interfacial contact thermal resistance [26], shape [27] and orientation distribution [28] of dispersion phase, etc. However, anisotropy of dispersed phase is still difficult to couple to MEA.

Despite that the macroscopic thermal conductivity of cast iron is isotropic, the contribution of graphite with different anisotropy is still unclear. The unit cell model can consider the anisotropy of the dispersed phase, which is a method to simplify the composites into a representative volume element (RVE) composed of phases with different thermal conductivity. The thermal conductivity of RVE, that is, the effective thermal conductivity of the composites, can be calculated by the thermal resistance network method [29]. Wang et al. [12] studied the effect of microstructure on the effective thermal conductivity of gray cast iron by establishing the unit cell model containing cross-shaped anisotropic graphite, which realized macroscopic isotropy in three main directions. It proved the feasibility of the unit cell model in the study of thermal conductivity of cast iron. To extend this approach to VGI, the complex shape of vermicular graphite needs to be simplified first, which can be solved by simple shape interpolation. Different growth rates of graphite along [1010] (a-axis) and [0001] (c-axis) determine the morphology of graphite. When the growth rate of the a-axis is much faster than that of the c-axis, it is easy to grow into flake graphite; when the growth rate of the a-axis is close to that of the c-axis, it is easier to grow into spherical graphite; while vermicular graphite is in between [30]. Therefore, if several simplified graphite models with topological equivalence [31,32] are established according to the 2-D and 3-D morphological characteristics of three types of graphite and the ratio of the a-axis and c-axis, it is believed that the real vermicular graphite will be included. Finally, the effect of interface between graphite and matrix is not also negligible on thermal conductivity of VGI [19,20].

In this study, a new theoretical method consisting of shape interpolation, unit cell model and numerical calculation was proposed. A series of unit cell models consisting of different simplified graphite models with topological equivalence and a hexahedron representing the matrix were established. Meanwhile, the virtual grids were used to represent the interface between graphite and matrix. Then, anisotropic simplified graphite models rotated in the matrix at full angles. The mathematical expectation of thermal conductivity at all angles, namely, the effective thermal conductivity of each unit cell model, was obtained by numerical calculation. The effects of microstructure on effective thermal conductivity of VGI were studied by the above method. The reliability of the calculated values of the models were further verified by the experimental data. This work can provide the basis for the effective regulation of thermal conductivity of VGI.

## 2. Methods

### 2.1. Computational Methods

Numerical calculation based on finite difference was used to calculate the effective thermal conductivity of unit cell model, which consisted of the simplified graphite model and a hexahedron representing the matrix. After the simplified graphite model rotated some angles in the matrix, the constructed unit cell model was meshed, and the grids were associated with parallel or series rule [33,34,35,36]. Thermal conductivity of unit cell model at this angle was calculated by the thermal resistance network method. Finally, mathematical expectation of thermal conductivity at all angles was effective thermal conductivity. The whole process was based on the implementation of visual studio community 2017 and written in C++ language. Further calculation details will be discussed below.

### 2.2. Experimental Methods

Three metallographic specimens taken from the same VGI RuT450 cylinder block were examined using the optical microscopy (LWD200-4XC). Before corrosion, the area and maximum centerline length of graphite were counted according to GB/T26656-2011. After the specimens were etched with 4% nital for 5–7 s, the relative content of pearlite in the matrix was counted. Each statistical value was the average of nine fields of view to eliminate the effect of uneven microstructure.

The thermal conductivity test specimens with dimensions of Φ12.5 mm × 2 mm were cut from the metallographic specimens using a linear cutting machine. The surface of every specimen was polished to ensure the reliability of thermal conductivity testing. The positive and negative sides of the sample were sprayed with graphite to increase the light energy absorption ratio and infrared emissivity of the sample surface. The sample density was set as 7.1 kg/m^3^. The thermal diffusivity was measured using a laser flash apparatus (Netzsch LFA457, Selb, Germany), the thermal conductivity was calculated according to λ = α·ρ·C_p, where α is the measured thermal diffusivity, ρ is the bulk density and C_p is the specific heat [37]. The thermal conductivity of each specimen was the average value of three measurements at 300.5 K (room temperature at test).

Finally, the round bars with dimensions of Φ1 mm × 15 mm were cut from the thermal conductivity test specimens. Three-dimensional morphology of graphite in the round bars was characterized using micro computed tomography (SkyScan2211). The accelerating voltage, the current and the precision were 80 kV, 190 μA and 300 nm, respectively. A total of 2357 projections were recorded for each specimen, with a size of 553.2 × 553.2 μm. After stacking reconstruction in sequence, the graphite volume fraction was counted.

## 3. Results and Discussion

### 3.1. Basis of Theoretical Model

Although the growth mechanism of graphite in cast iron has not yet formed a unified understanding, the empirical method of controlling graphite morphology is mature. Flake graphite can be transformed into vermicular graphite and then into spherical graphite by adding a small amount of spheroidized elements, such as Mg, Ce or lanthanide. This can be explained roughly from the preferred growth direction of graphite. Each monolayer along the a-axis (perpendicular with [0001] direction) of graphite is a 2-D polymeric graphene sheet bonded by a strong covalent, and all monolayers along the c-axis (parallel with [0001] direction) of graphite are bonded by weak van der Waals force [38]. Therefore, graphite preferentially grows along the a-axis to flake graphite without adding spheroidized elements. With the increase in spheroidized elements, graphite morphology gradually transforms into a spherical shape [30]. Our previous work [39] also confirmed the direct interaction between the adding spheroidized elements and graphite phase during growth process by combining first-principles calculation and TEM analysis. Meanwhile, different bonding modes of graphite along a-axis and c-axis lead to strong anisotropy of graphite thermal conductivity. Consequently, different morphology of graphite has different thermal conductivity, mainly due to the anisotropy caused by the ratio of a-axis and c-axis.

**Figure 1:**
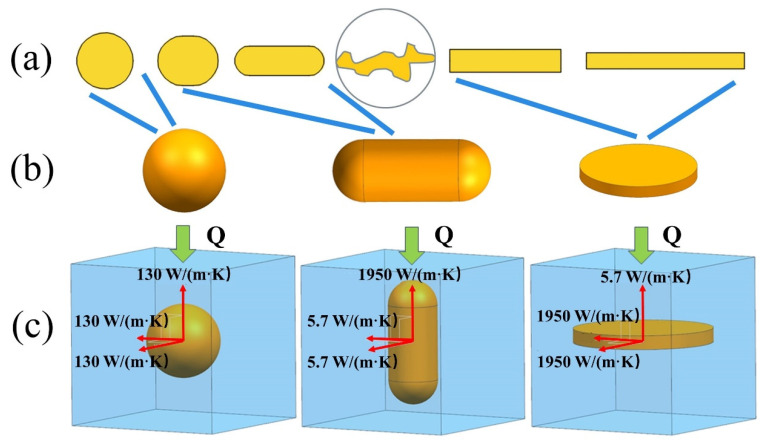
Establishment of a series of unit cell models by shape interpolation. (**a**) Two-dimensional abstractions of typical structures in metallographic photographs. (**b**) Three-dimensional abstractions of typical structures in metallographic photographs. (**c**) Demonstrations about method to calculate equivalent thermal conductivity (one of all directions) of three-dimensional abstractive models.

As shown in Figure 1a, several 2-D simplified graphite models with different shapes were established based on the ratio of a-axis and c-axis. According to the growth rules of graphite described above, the capsule-based shape was used to transition between a circle representing spherical graphite and slender rectangles representing flake graphite. The real 2-D morphology of vermicular graphite was considered to be included. In space, spherical graphite particles are spherical, and flake graphite particles presents the appearance of relatively flat sheets connected together [20,40]. In addition, vermicular graphite particles are coral-like and each branch is curved cylinder with round head [20]. Therefore, 3-D simplified graphite models including spherical, capsule-based and disc type were established according to the 3-D morphological characteristics of three types of graphite, as shown in Figure 1b. The graphite in VGI with the vermicularity of 0–100% is considered to be between the spherical and disc type.

Figure 1c shows the series of unit cell models consisting of different simplified graphite models and a blue hexahedron representing the matrix. Then, different thermal conductivity was assigned to the a-axis and c-axis of each simplified graphite model. For anisotropic graphite in cast iron, the thermal conductivity of the a-axis and c-axis are usually considered to be 1950 W/(m·K) and 5.7 W/(m·K), respectively [39]. Meanwhile, spherical graphite is usually regarded as isotropic, and the thermal conductivity is considered to be 130 W/(m·K) [16,34]. The same values were used in this paper. The blue hexahedron representing the matrix remained stationary in the world coordinate system (WCS), and the direction of heat flux was fixed to the *z*-axis of WCS. The a-axis and c-axis with different thermal conductivity were bound with the simplified graphite models and rotated together in the matrix. At present, experiments have confirmed that the spheroidal graphite in nodular cast iron is actually formed by the polymerization of curved flaky graphite [35]. So, it is more reasonable to assume that spheroidal graphite has isotropic thermal conductivity rather than anisotropic, the thermal conductivity of spheroidal graphite in this paper cannot be calculated directly using anisotropic graphite. Therefore, we finally selected 130 W/m·K as the reference value for calculation.

**Figure 2:**
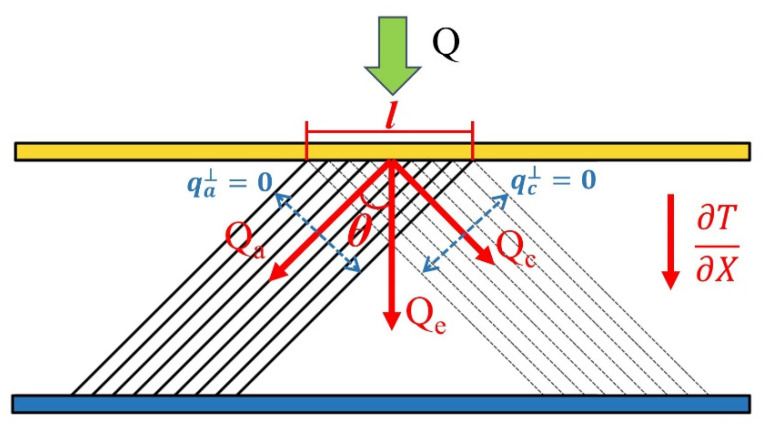
Conversion of non-main direction thermal conductivity of graphite under the one-dimensional steady heat transfer.

The a-axis and c-axis of anisotropic graphite are the two main directions of heat transfer. Under one-dimensional steady heat transfer, the heat transfer in the direction of heat flow can be regarded as the vector sum of the heat transfer of the a-axis and the c-axis. Figure 2 shows the conversion of thermal conductivity in non-main direction. Assume that heat is only transferred along the a-axis and c-axis, and the direction perpendicular to a-axis or c-axis is adiabatic. Then, the temperature gradients along the a-axis and c-axis are $\partial T/\partial \left(\frac{X}{\cos\theta }\right)$ and $\partial T/\partial \left(\frac{X}{\sin\theta }\right)$, respectively. According to Equations (1)–(4), the solution of effective thermal conductivity along the *z*-axis of WCS was determined.

(1) $$Q_{e}={\;Q}_{a}+Q_{c}$$

(2) $$q_{e}l\;={\;q}_{a}l\cos\theta +q_{c}l\sin\theta$$

(3) $$\lambda_{e}\frac{\partial T}{\partial X}l=\lambda_{a}\frac{\partial T}{\left(\partial X/\cos\theta \right)}l\cos\theta +\lambda_{c}\frac{\partial T}{\left(\partial X/\sin\theta \right)}l\sin\theta$$

(4) $$\lambda_{e}=\lambda_{a}*\cos^{2}\theta +\lambda_{c}*\sin^{2}\theta$$

where Q, q and λ are heat, heat flux and thermal conductivity, respectively, the subscripts e, a and c represent the direction along the heat flux (equivalent direction), a-axis and c-axis of graphite, respectively and θ is the angle between the a-axis of graphite and the direction along the heat flux. This is the main part of the equivalent thermal conductivity program written in C++ in our study.

The interface between graphite and matrix (hereinafter referred to as interface) was represented by the virtual grids wrapped on the surface of each simplified graphite model. The assignment of thermal conductivity and thickness of virtual grids in each unit cell model was consistent. In some theoretical calculation researches on the interface between graphite and metal [36,41], the modeling thickness is nm order of magnitude. Although in cast iron, the thickness of interface may be greater than this magnitude. As a reasonable approximation of the theoretical method, it is feasible to assign the thickness of the virtual grids representing the interface to 10^−9^ m. Then, the assignment of the thermal conductivity of the virtual grids was used to make the numerical calculation results close to the actual values. The range of graphite volume fraction, thermal conductivity of matrix and nodular cast iron is usually 5–10%, 25–40 W/(m·K) and 25–40 W/(m·K), respectively [14,42]. Table 1 shows the calculated values of nodular cast iron corresponding to different assignments of thermal conductivity of the virtual grids. The final assignment adopted in this paper was 10^−6^ W/(m·K), which also covered the effects of the actual thickness and incomplete contact of the interface, crystal defects, etc., on the heat conductivity of cast iron.

Where *v*, $\lambda_{I}$, $\lambda_{m}$ and $\lambda_{g}$ are graphite volume fraction and thermal conductivity of interface, matrix and isotropic spherical graphite, respectively.

Before the numerical calculation, a vector direction (*φ*,*θ*) was defined by using the dual-angle method of polar coordinates, where the direction was *z*-axis of WCS when *φ* = 0, and the direction is *x*-axis of WCS when *φ* = π/2 and *θ* = 0. A spherical surface with radius of 1 was used to represent the probability of each vector appearing. Since the calculation of the positive and negative directions of thermal conductivity is consistent and the hemisphere is inverse symmetric, the value range of *φ*∈[0,π/2] and *θ*∈[0,2π] was sufficient to cover all angles of graphite rotation. ΔS was calculated by Equation (5), representing the area of the micro-element on the spherical surface corresponding to each step of the graphite rotation. Therefore, the mathematical expectation of thermal conductivity at all angles, that is, the effective thermal conductivity of each unit cell model, could be calculated by Equation (6). Finally, the parameters adopted in numerical calculation were shown in Table 2.

(5) ΔS=sinφΔφΔθ

(6) $$\lambda_{e}=\left(\underset{\begin{array}{c} 0\leq \;\varphi \;\leq \;\pi /2\\ 0\leq \;\theta \;\leq \;2\pi \; \end{array}}{\sum }\lambda_{\left(\varphi ,\;\theta \right)}\sin\varphi \right)/2\pi$$

where $\lambda_{e}$ is effective thermal conductivity of unit cell model and $\lambda_{\left(\varphi ,\;\theta \right)}$ is thermal conductivity of unit cell model when graphite rotated to (φ, θ).

### 3.2. Effects of Microstructure on Effective Thermal Conductivity

Generally, the volume fraction and shape of graphite and thermal conductivity of matrix are the main factors affecting the thermal conductivity of cast iron [14]. In the discussion above, the influence of anisotropy of graphite and interface cannot be ignored as well. The effects of these factors on thermal conductivity of VGI were discussed below. Moreover, it is difficult to propose a parameter to accurately describe the 3-D morphology of vermicular graphite and separate independent vermicular graphite particles in space. RSF proposed by GB/T26656-2011, which represents the ratio of graphite area to its circumscribed circle area, can well distinguish the 2-D morphology of graphite. Graphite shape parameter *k* was defined in this paper to describe the 3-D morphology of graphite, which could be calculated by $k={\left(\sqrt{RSF}\right)}^{3}$. To make the model more usable in real manufacture situations, we chose shape characteristic factors from a 2-D metallographic model rather than a 3-D model. However, every value was the average from nine metallographic datapoints to confirm the result will not be influenced by local uneven microstructure.

#### 3.2.1. Anisotropy of Graphite and Interface between Graphite and Matrix

**Figure 3:**
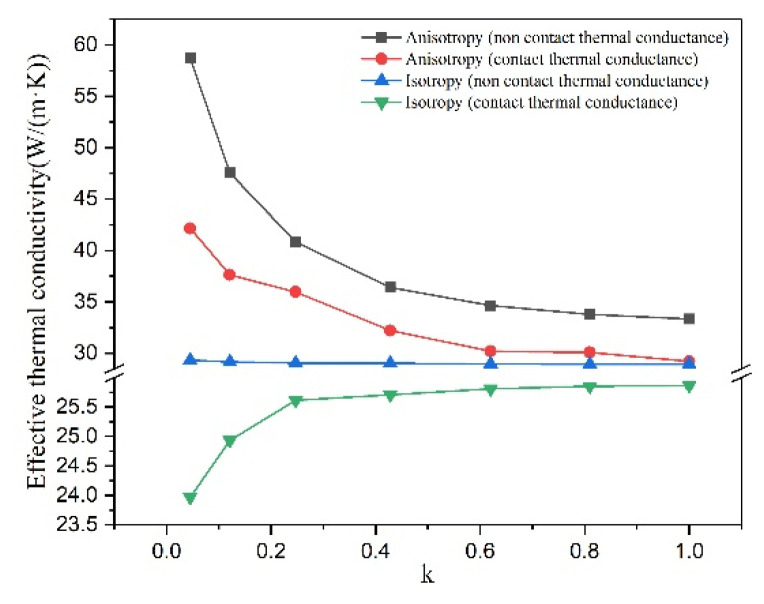
Effects of graphite anisotropy and ICTC on effective thermal conductivity of unit cell models containing simplified graphite models with different shape, *v* = 5%, $\lambda_{m}$ = 25 W/(m·K).

Figure 3 shows the effects of graphite anisotropy and ICTC on effective thermal conductivity of unit cell models containing simplified graphite models with different shape, where *v* is 5% and $\lambda_{m}$ is 25 W/(m·K). It can be seen that the effective thermal conductivity is independent of the graphite shape when the graphite is isotropic and the ICTC is not considered (blue line). The green line, considering graphite isotropy and ICTC, indicates that the effective thermal conductivity decreases with the graphite deviates from the sphere. This is because the surface area of graphite is equivalent to the interface area, which increase enhances the hindrance effect of the interface on the effective thermal conductivity. The black line, considering graphite anisotropy (involving spherical type) and non-ICTC, shows effective thermal conductivity increases with graphite shape deviating from sphere due to the enhancement of graphite anisotropy. The red line, considering graphite anisotropy (involving spherical type) and ICTC, changes more gently than the black line. The same change trend of red line and black line reveals that the more the graphite shape deviates from the sphere, the greater the enhancement effect of graphite anisotropy on thermal conductivity than the hindrance effect of interface. It is noteworthy that these two effects are close when the graphite is spherical type. In addition, the following analysis of microstructure effects is based on considering graphite anisotropy and ICTC.

#### 3.2.2. Graphite Shape Parameter

**Figure 4:**
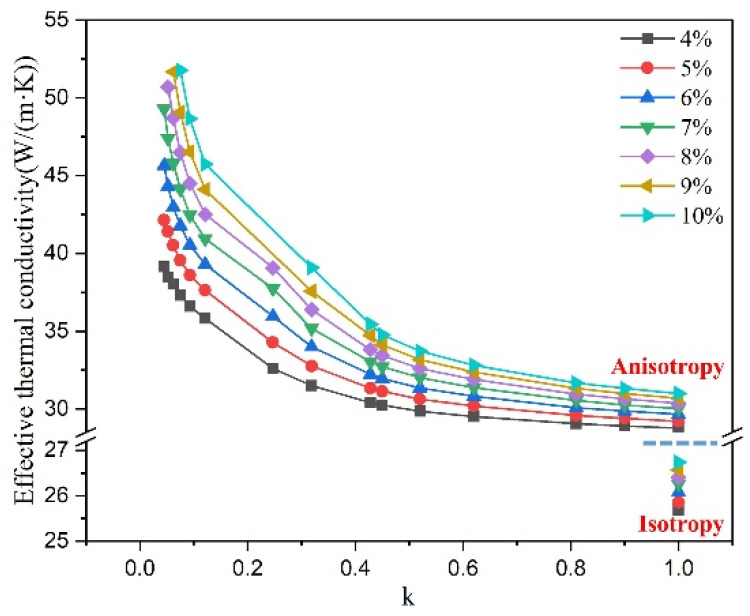
Effect of graphite shape parameter on effective thermal conductivity of cast iron, $\lambda_{m}$ = 25 W/(m·K) and spherical type: k = 1; capsule-based type: 0.9 > k > 0.24; disc type: 0.12 > k > 0.04.

Figure 4 shows the effect of graphite shape parameter on effective thermal conductivity of cast iron, where $\lambda_{m}$ is 25 W/(m·K). The effective thermal conductivity of cast iron increases in the form of a power function with the decrease of the shape parameter, which is the same as the rule revealed by the red line in Figure 3. Furthermore, the relatively smooth curves show that the three types of constructed simplified graphite models have a good transition relationship. 

#### 3.2.3. Graphite Volume Fraction

**Figure 5:**
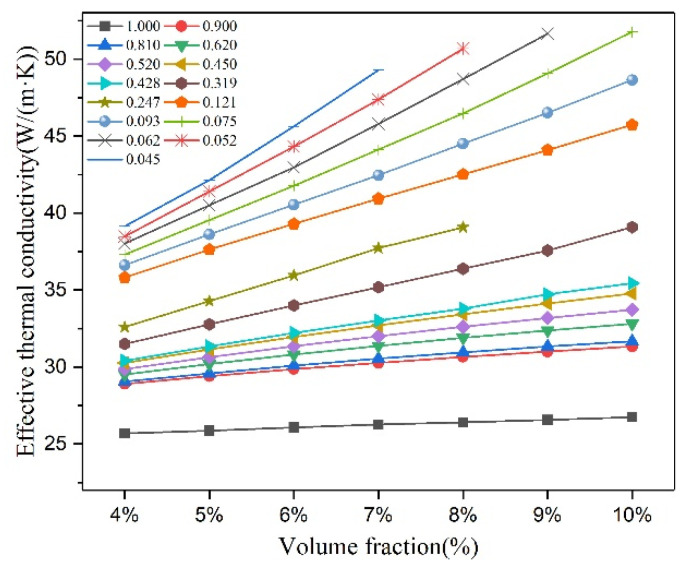
Effect of graphite volume fraction on effective thermal conductivity of cast iron, $\lambda_{m}$ = 25 W/(m·K).

Figure 5 shows the effect of graphite volume fraction on effective thermal conductivity of cast iron, where $\lambda_{m}$ is 25 W/(m·K). It can be seen that with the graphite volume fraction increases, the effective thermal conductivity of cast iron increases linearly. Meanwhile, the smaller the graphite shape parameter, the more drastic the change. This is because with the decrease in the graphite shape parameter, the anisotropy of graphite increases and the heat transfer along a-axis increases, resulting in an increase in the thermal conductivity of graphite. Graphite is the reinforcing phase of heat conduction in cast iron. When the difference between the thermal conductivity of graphite and that of matrix increases, the effective thermal conductivity of cast iron is more sensitive to the change in graphite volume fraction.

#### 3.2.4. Thermal Conductivity of Matrix

**Figure 6:**
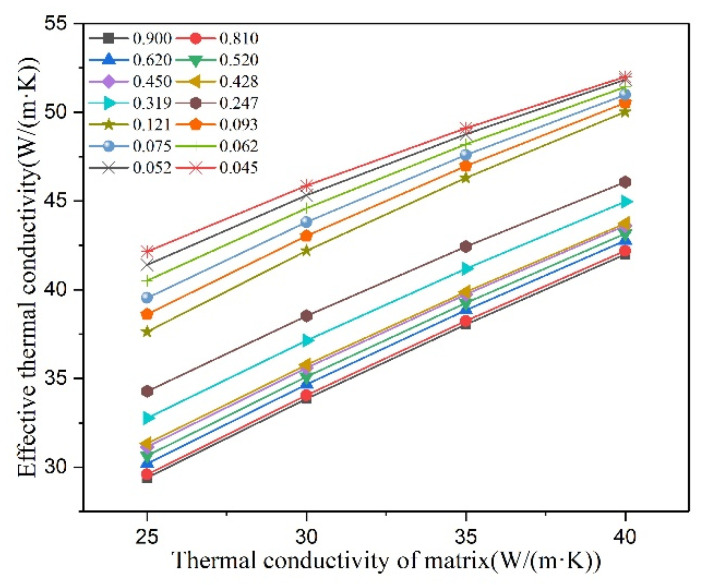
Effect of thermal conductivity of matrix on effective thermal conductivity of cast iron, *v* = 5%.

Figure 6 shows the effect of thermal conductivity of matrix on effective thermal conductivity of cast iron, where *v* is 5%. The effective thermal conductivity of cast iron increases linearly with the thermal conductivity of matrix increases. At the same time, the slope of linear change decreases with the decrease of graphite shape parameter, which corresponds to the analysis in Section 3.2.3.

### 3.3. Establishment of Quantitative Model and Verification

The effect of microstructure on thermal conductivity of VGI was discussed in detail above. In order to further verify the reliability of the model, quantitative calculation and experiments were necessary. Firstly, all microstructure characteristics need to be unified into a formula. A near linear relationship is displayed between the thermal conductivity of matrix and effective thermal conductivity of cast iron in Figure 6. For the unit cell model composed of graphite and matrix, it is obvious that the slope and intercept of the near linear relationship are affected by the thermal conductivity of graphite. Meanwhile, the above analysis demonstrates that the thermal conductivity of graphite depends on the volume fraction and shape of graphite. Therefore, the near linear relationship can be expressed by Equation (7), where the f(v, k) and g(v, k) were obtained by polynomial fitting, respectively, as shown in Equations (8) and (9).

(7) $$\lambda_{e}(k,\;v,\;\lambda_{m})=f\left(v,\;k\right)\lambda_{m}+g(v,\;k)$$

$$f\left(k,\;v\right)=1.052+0.1788k\;-\;8.467v\;-\;{1.161k}^{2}+15.86kv+{14.06v}^{2}+{0.8992k}^{3}$$

(8) $$-{8.635k}^{2}*v\;-\;{24.65kv}^{2}\;\mathrm{and}\;R^{2}=0.92;\;\mathrm{Adjusted}\;R^{2}=0.91$$

(9) $$\begin{array}{r} g\left(k,\;v\right)=-270.13+{501.24k}^{-0.36}\;+{249.56v}^{-0.0251}\;-\;{456.62k}^{-0.36}v^{-0.0251}\\ \;\mathrm{and}\;R^{2}=0.98;\;\mathrm{Adjusted}\;R^{2}=0.98 \end{array}$$

**Figure 7:**
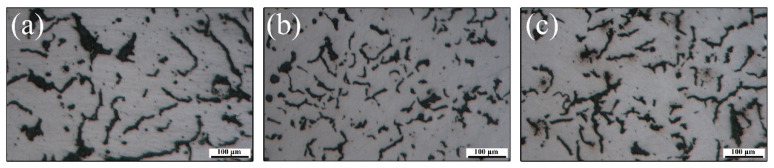
Two-dimensional morphology of graphite in (**a**) specimen 1, (**b**) specimen 2 and (**c**) specimen 3 [43].

**Figure 8:**
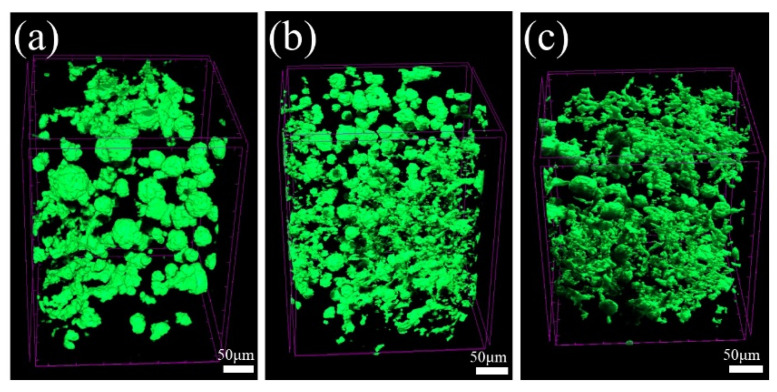
Three-dimensional morphology of graphite in (**a**) specimen 1, (**b**) specimen 2 and (**c**) specimen 3.

Figure 7 shows the 2-D morphology of graphite in three specimens taken from the same cylinder block. It can be seen that although the composition is consistent, there are some differences in graphite morphology due to different cooling rates. The graphite in specimen 1 is relatively straight and slender, and average areas of all graphite particles is obviously larger than that of specimen 2 and 3. The graphite in specimen 3 is almost all vermicular graphite as well. However, several spherical graphite appears in specimen 2, which will be reflected in graphite shape parameter *k*. The corresponding 3-D morphology of graphite in three specimens is shown in Figure 8. It is apparent that the average 3-D size of graphite is related to the average areas of all graphite particles in Figure 7. Moreover, it is found that there is no good correspondence between the total area of graphite particles in 2-D and the total volume in 3-D by quantitative statistics. This also proves the necessity of building 3-D model to research the thermal conductivity of VGI. The pearlite fraction of the three specimens is all about 92%. Therefore, the thermal conductivity of matrix was assigned to 25 W/(m·K). The calculation results are shown in the Table 3, and the error is about 2%. Metallographic images in Figure 7 and experimental values of thermal conductivity in Table 3 are derived from previous work [43].

In addition, Liu et al. [44] studied the effect of graphite morphology on mechanical properties and thermal conductivity of VGI by micro-CT test. Metallographic images, graphite volume fraction and thermal conductivity were provided in this literature. We calculated graphite shape parameter *k* according to metallographic images in literature. Due to the thermal conductivity of the matrix was not shown, the thermal conductivity of the matrix was inversely calculated by the quantitative model established in this paper. The results were $\lambda_{m,(a)}$ = 6.89 W/(m·K), $\lambda_{m,(b)}$ = 7.35 W/(m·K), $\lambda_{m,(c)}$ = 5.97 W/(m·K) and $\lambda_{m,(d)}$ = 7.86 W/(m·K), respectively. The thermal conductivity of matrix was assigned to the average value, that is, 7.02 W/(m·K). Finally, the calculation results are shown in the Table 4, and the error is about 3%. Graphite skeleton structure with spatial connectivity is generally considered to be an important factor in improving the thermal conductivity of cast iron [16]. However, by our studies, we suspect that the underestimation of thermal conductivity may be more due to whether the anisotropy of graphite’s heat conduction is considered. In future work, we will construct a larger scale graphite 3D network model to systematically study the impact of connectivity.

## 4. Conclusions

By combining shape interpolation, unit cell model and numerical calculation, a new theoretical method was successfully applied to the study of thermal conductivity of VGI. It overcame the tough complex morphology of vermicular graphite and systematically studied the effect of graphite anisotropy and ICTC on thermal conductivity in addition to conventional phase proportion and shape. The reliability of the model was verified by experimental data. The main conclusions are listed as follows:

(1) The effective thermal conductivity of VGI increases in power function with the decrease in graphite shape parameter, and increases linearly with the increase in graphite volume fraction and thermal conductivity of matrix.
(2) The change in graphite shape affects both graphite anisotropy and area of interface. The more the graphite shape deviates from the sphere, the greater the enhancement effect of graphite anisotropy on thermal conductivity than the hindrance effect of interface. These two effects are close when the graphite is spherical type.
(3) A quantitative model was built to predict the effective thermal conductivity of VGI, which was suitable for the vermicularity of 0–100%.
(4) The experimental results are in good agreement with the model calculations, and the error is about 3%. This proves the rationality of the models and assumptions adopted in the theoretical method.

## Figures and Tables

**Table 1:** The calculated values of nodular cast iron corresponding to different assignments of thermal conductivity of the virtual grids. Unit: W/(m·K).

	$\lambda_{I}=10^{-5}$	$\lambda_{I}=10^{-6}$	$\lambda_{I}=10^{-7}$
*v* = $5\%,\;\lambda_{m}$ $=25,\;\lambda_{g}$ = 130	28.60	25.86	13.20
*v* = $10\%,\;\lambda_{m}$ $=40,\;\lambda_{g}$ = 130	45.87	36.17	11.61

**Table 2:** Parameters of numerical calculation.

Parameters	Valuation
Size of unit cell model	80 × 80 × 80
Number of calculation angles	1061
Steps of mesh generation	100 × 100 × 100
Graphite volume fraction	*v* = 4–10%
Thermal conductivity of isotropic graphite (spherical type)	$\lambda_{g}$ = 130 W/(m·K)
Thermal conductivity of anisotropic graphite (capsule-based type and disc type)	$\lambda_{a}$$=1950\;W/(m\cdot K),\;\lambda_{c}$ = 5.7 W/(m·K)
Thermal conductivity of matrix	$\lambda_{m}$ = 25–40 W/(m·K)
Thermal conductivity of interface	$\lambda_{I}$ = 10^−6^ W/(m·K)
Thickness of interface	T = 10^−9^ m

**Table 3:** The comparison of experimental value and predicted value of own data.

553 × 553 × 707 μm^3^	v	k	$\lambda_{m}$ W/(m·K)	Experimental Value [43] W/(m·K)	Predicted Value W/(m·K)	Error %
specimen 1	0.060	0.145	25	38.904	39.689	2.017
specimen 2	0.042	0.150	25	34.951	35.737	2.248
specimen 3	0.043	0.145	25	36.095	36.144	0.135

**Table 4:** The comparison of experimental values and predicted values of literature data.

599 × 599 × 839 μm^3^	v	k	$\lambda_{m}$ W/(m·K)	Experimental Value W/(m·K)	Predicted Value W/(m·K)	Error %
(a)	0.0998	0.428	7.02	23.65	23.75	0.42
(b)	0.0999	0.407	7.02	24.47	24.20	1.10
(c)	0.1029	0.250	7.02	28.16	29.04	3.13
(d)	0.1032	0.246	7.02	29.95	29.24	2.37

## Data Availability

Data will be made available on request.
